# Magnetic resonance imaging findings in pseudo-Meigs' syndrome associated with a large uterine leiomyoma: a case report

**DOI:** 10.1186/1752-1947-4-120

**Published:** 2010-04-28

**Authors:** Danai Chourmouzi, Elissavet Papadopoulou, Antonios Drevelegas

**Affiliations:** 1 Interbalkan Medical Center, Asklipiou, Pylaia 57001, Thessaloniki, Greece

## Abstract

**Introduction:**

Pseudo-Meigs' syndrome is a rare pathological entity characterized by the presence of a pelvic mass other than an ovarian fibroma. The mass is associated with ascites with or without hydrothorax.

**Case presentation:**

We describe the case of a 41-year-old Caucasian woman with a large uterine leiomyoma associated with massive ascites. A magnetic resonance imaging scan showed a large subserosal leiomyoma with multiple areas of cystic degeneration.

**Conclusion:**

To the best of our knowledge, this is the first reported case of pseudo-Meigs' syndrome caused by a uterine leiomyoma and diagnosed using magnetic resonance imaging. The pathophysiology of this syndrome and the role of magnetic resonance imaging are emphasized in this case report.

## Introduction

Pseudo-Meigs' syndrome is a rare pathological entity characterized by the presence of a pelvic mass other than an ovarian fibroma. The mass is associated with ascites with or without hydrothorax.

We report the case of a patient with pseudo-Meigs' syndrome caused by a uterine leiomyoma. A magnetic resonance imaging (MRI) scan provided a detailed description of the tumor. Our patient's MRI results correlated well with histological findings and helped us make a diagnosis, which enabled the avoidance of a hysterectomy. To the best of our knowledge, currently fewer than 35 cases of pseudo-Meigs' syndrome have been reported in the literature and our case of pseudo-Meigs' syndrome is the first to be diagnosed using MRI.

## Case presentation

A 41-year-old Caucasian woman presented at our hospital with a 12-month history of abdominal swelling, discomfort, urinary frequency and incontinence. She had regular menstrual cycles and had never been pregnant. Her clinical examination revealed a marked distension of her abdomen and a large palpable mass in her central pelvis. Gynecological vaginal ultrasound (US) showed abundant ascites in her pelvis, as well as a solid, smoothly outlined mass with heterogenous echogenicity. The mass seemed to extend from her pelvic cavity to her abdomen on the midline above the uterus. Her uterus and ovaries could not be identified separately from the pelvic mass. The mass was considered to be of adnexal origin. Laboratory test results of our patient showed the following values: serum carbohydrate antigen (CA)-125 level at 436.7 U/mL (normal value < 30 U/mL), fetoprotein (FP) at 2.8 ng/mL, (normal value<10 ng/ml) beta-human chorionic gonadotropin (β-HCG) at 5.0 mIU/mL (normal value < 3), and carcinoembryonic antigen (CEA) 1.07 ng/mL (normal value < 5 ng/ml). An MRI scan was requested to further evaluate our patient and to determine the exact nature of her mass.

**Figure 1:**
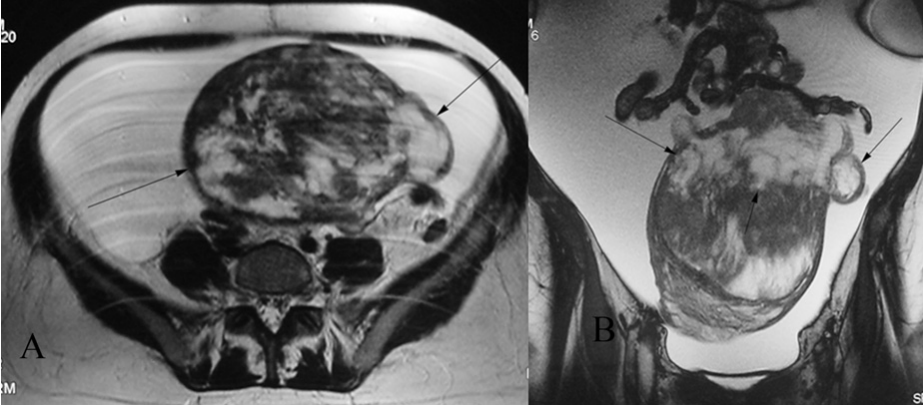
**(A) Axial and (B) coronal T2-weighted fast spin echo magnetic resonance images which show a large ovoid subserosal leiomyoma in the pelvis that extends superiorly from the body and fundus of the uterus**. The mass is heterogeneous with predominantly low to intermediate signal intensity relative to that of the outer myometrium. Several small foci of very high signal intensity are also seen (arrows). A normal endometrial stripe and junctional zone are seen. Note the presence of massive ascites.

**Figure 2:**
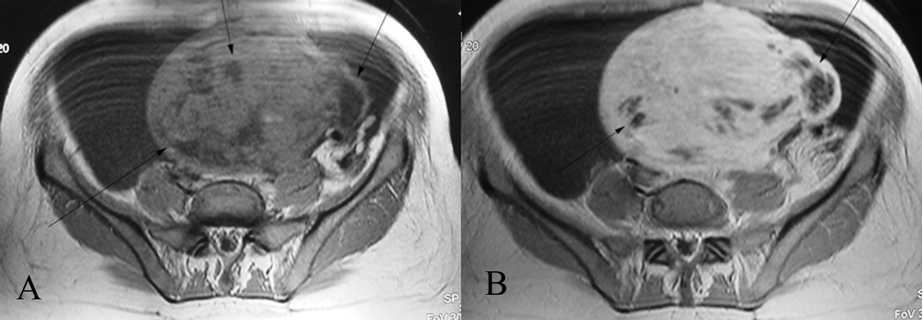
**(A) Axial T1-weighted spin echo and (B) contrast-enhanced axial T1-weighted spin echo magnetic resonance image which show similar enhancement of the normal myometrium and mass**. The foci of high signal intensity on the T2-weighted image show no enhancement on the contrast-enhanced images representing areas of cystic degeneration (arrows).

The results of our patient's MRI scan revealed massive ascites and a heterogeneous ovoid pelvic mass measuring 13 × 16 cm. The mass had a broad connection to the uterus. It was located subserosally and extended superiorly from the posterior body and fundus of her uterus. The uterus was displaced inferiorly. She was noted to have a normal endometrial stripe, a normal junctional zone, and normal ovaries. The mass was heterogeneous and produced a predominantly low to intermediate signal intensity on T2-weighted images relative to that of the outer myometrium. Several small foci with very high signal intensities were also seen (Figure 1). The foci of high signal intensity on the T2-weighted images had low signal intensity on the T1-weighted images, and they showed no enhancement on the contrast-enhanced images, representing areas of cystic degeneration (Figure 2). Based on the above imaging findings, the diagnosis of a large subserosal leiomyoma with areas of cystic degeneration was made. We recommended excision of the mass without hysterectomy.

**Figure 3:**
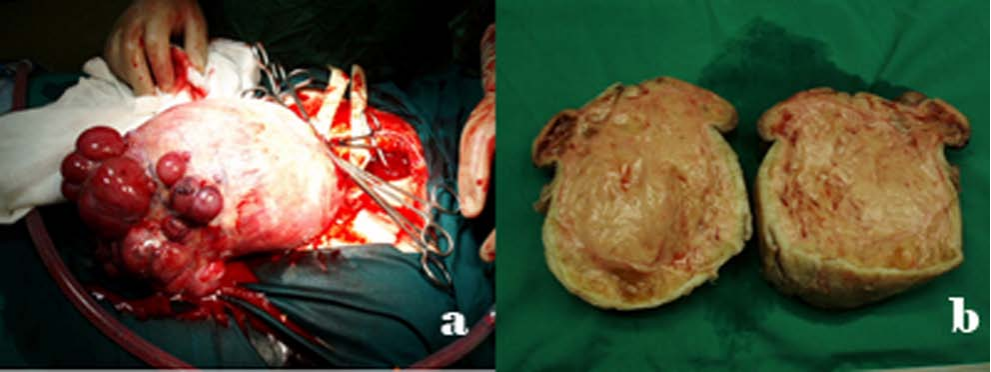
**(A) Surgical specimen during the operation which shows a large solid mass with multiple grapelike projections corresponding to the cystic areas seen on magnetic resonance**. (B) Serous fluid was seen pouring from multiple cysts on cutting the tumor.

Our patient underwent an exploratory laparotomy. A large firm mass, which originated from the uterine fundus, was seen. Multiple lobulated projections were also seen on the superior border of the mass (Figure 3). The mass was then excised and the ascitic fluid was drained. Histopathological examination of the mass revealed the presence of a uterine leiomyoma. Our patient had an uneventful post-operative course.

## Discussion

Pseudo-Meigs' syndrome is characterized by the presence of ascites. It is also often characterized by pleural effusion caused by a pelvic tumor other than an ovarian fibroma. Tumors associated with pseudo-Meigs' syndrome are usually found in women's genitalia. The most commonly described tumor type is a leiomyoma, which is usually found in the uterus or the broad ligament [1]. Other reported ovarian tumors responsible for pseudo-Meigs' syndrome are struma ovarii tumors, mucinous or serous cystadenomas, germ cell tumors and ovarian metastasis from colon and stomach cancers [2].

Uterine leiomyomas are the most commonly reported cause of pseudo-Meigs' syndrome. They usually manifest as increased abdominal distension caused by a progressively enlarging pelvic mass and ascites. Respiratory insufficiency caused by pleural effusion is also often encountered [3].

It is speculated that the presence of ascites results from mechanical irritation of the peritoneum and the leakage of intratumoral fluid from the degenerated leiomyoma. As leiomyomas enlarge, they may outgrow their blood supply, thus resulting in various types of degeneration.

Cystic degeneration is considered to be a sequela of edema and is observed in about 4% of reported cases of leiomyomas. Cystic spaces appear as round, well-demarcated areas with signal intensities that have the characteristics of fluid, namely low on T1-weighted images and high on T2-weighted images with no enhancement [4]. Multiple lobulated, fluid-filled, grape-like cystic areas were seen in our patient. These cystic areas projected from the superior border into the peritoneal cavity which was presumed to be the cause of her massive ascites.

Pleural effusions, which are commonly right-sided, result from transdiaphragmatic transport of ascitic fluid [5]. Although our patient had massive ascites, no pleural effusion was detected.

Laboratory tests usually reveal an elevated serum CA-125 level caused by peritoneal irritation [5,6]. Our patient had a serum CA-125 level of 436.7 U/mL, the normal value being <30 U/mL.

An MRI scan enables the detection and characterization of leiomyomas, as well as their differentiation from other types of adnexal masses. If MRI can demonstrate the continuity of an adnexal mass to adjacent myometrium then a diagnosis of leiomyoma can be established. The ability of MRI to visualize normal ovaries, even in the presence of an enlarged, myomatous uterus, may aid in determining the origin of the pelvic masses by excluding a diagnosis of ovarian neoplasm [7].

Ovarian fibromas and Brenner tumors are benign ovarian neoplasms that have a large fibrous component. These neoplasms can have signal intensities similar to those of pedunculated leiomyomas. MRI can show fibromas and Brenner tumors surrounded by ovarian stroma and follicles, thus establishing the ovarian origin of the mass and excluding a diagnosis of leiomyoma [8].

MRI has been shown to be more sensitive than US in detecting leiomyomas. An accurate assessment of an enlarged, myomatous uterus is not consistently possible with US because of its limited field of view.

Resection of the tumor leads to the resolution of the ascites and pleural effusion, therefore a thorough knowledge of the pseudo-Meigs' syndrome is important. Although the concomitant existence of a pelvic mass, ascites and pleural effusion is highly indicative of malignancy, hysterectomy and bilateral salpingo-oophorectomy can be avoided.

## Conclusion

Pseudo-Meigs' syndrome is a well-recognized pathological entity characterized by the coexistence of a pelvic mass, other than an ovarian fibroma, with ascites and hydrothorax. MRI is highly sensitive in detecting the relationship of a tumor with its adjacent structures and in providing sufficient correlation with histological findings. MRI thus renders a correct diagnosis possible, so that aggressive surgical treatment can be avoided.

## Consent

Written informed consent was obtained from the patient for publication of this case report and any accompanying images. A copy of the written consent is available for review by the Editor-in-Chief of this journal.

## Competing interests

The authors declare that they have no competing interests.

## Authors' contributions

DC and EP analyzed and interpreted our patient data and were major contributors in writing the manuscript. AD analyzed our patient data and contributed in writing the manuscript. All authors read and approved the final manuscript.
